# Thermo-sensitive self-assembly of poly(ethylene imine)/(phenylthio) acetic acid ion pair in surfactant solutions

**DOI:** 10.1080/10717544.2022.2027571

**Published:** 2022-07-09

**Authors:** Fanyu Zhao, Jin-Chul Kim

**Affiliations:** Department of Biomedical Science & Institute of Bioscience and Biotechnology, Kangwon National University, Chuncheon 24341, Republic of Korea

**Keywords:** Ion pair, oxidation, (Phenylthio) acetic acid, surfactant, ionic nanoparticle

## Abstract

Poly(ethylene imine)/(phenylthio) acetic acid (PEI/PTA) ion pairs exhibited an upper critical solution temperature (UCST) behavior in an aqueous solution and the UCST was higher as the PTA content was more. The UCST of the ion pair decreased with increasing Brij S100 (BS 100, a nonionic surfactant) concentration but increased with increasing cetylpridinium chloride (CPC, a cationic surfactant) and sodium lauroylsarcosinate (SLS, an anionic surfactant) concentration. TEM microscopy demonstrated BS 100 markedly reduced the size of PEI/PTA ion pair self-assembly (IPSAM) whereas CPC and SLS had little effect on the size and the integrity of IPSAM. ^1^H NMR spectroscopy showed the hydrophobic interaction among the phenyl groups of PEI/PTA ion pairs took place. It also demonstrated the hydrophobic interaction between BS 100 and PTA and the electrostatic interaction between CPC and PTA and between SLS and PEI occurred. X-ray photoelectron spectroscopy disclosed the PTA of PEI/PTA IPSAM could be readily oxidized by H_2_O_2_ even at a low concentration (e.g. 0.005%). IPSAM released its payload (i.e. nile red) in a temperature and oxidation-responsive manner. The surfactants (i.e. BS 100, CPC, and SLS) suppressed the thermally triggered release in a different way. The effectiveness of the surfactant to suppress the release was in the order of BS 100 > CPC > SLS. IPSAM released its content more extensively as H_2_O_2_ (an oxidizing agent) concentration was higher. The ionic surfactants (i.e. CPS and SLS) had little effect on the oxidation-induced release degree but the nonionic surfactant (BS 100) markedly suppressed the release degree.

## Introduction

The self-assembly of amphiphilic molecules is spontaneously formed in an aqueous solution via an entropy-driven process (Tanford, 1973; Israelachvili et al., 1980; Michel & Cleaver, 2007; Sorrenti et al., 2013). A major determinant for the shape and the structure of the self-assembly is the shape of amphiphilic molecules because amphiphilic molecules act as the building block and constitute the assembly following a space-filling model. The shape of amphiphilic molecules can be characterized by the packing parameter (P) (Tanford, 1973; Khalil & Zarari, 2014; Lombardo et al., 2015; Doncom et al., 2017; Lombardo et al., 2020). If the packing parameter is around 1, an amphiphilic molecule is rectangular and it can build up bilayer. If the packing parameter is much different from 1, an amphiphilic molecule is conical (*P* < 1) or reversed conical (*P* > 1) and it can be assembled into micelles or hexagonal phase (Tanford, 1973; Sych et al., 2018; Sagnella et al., 2010; N. Wang et al., 2019). Much attention has been paid to self-assembly for its use as a drug carrier because it shows the versatile property in several aspects.

The method of preparation is simple and easy and it seldom involves the use of an organic solvent that may do harm the human body. Only the simple dispersion of an amphiphilic molecule in an aqueous solution leads to the formation of the self-assembly, once the concentration is above the critical micellization concentration, defined as the concentration of surfactants above which micelles form (Tanford, 1973; Bhattarai et al., 2017). Owing to the amphiphilic property of the building block, there are both the hydrophilic and the lipophilic compartment in the self-assembly. As a result, the assembly can accommodate compounds with a broad spectrum of polarity (Skrabania et al., 2010; Yang et al., 2018). The self-assembly is inherently nano-sized or easily made to be so small with aid of the micronizing energy and a colloid stabilizer (i.e. surfactants). They readily penetrate into biological tissues due to their small size and can be targeted to and internalized into cells if the surface is decorated with an adequate ligand (Blanco et al., 2015; Din et al., 2017; Lombardo et al., 2019). The self-assembly can be designed to exhibit a responsive release to stimuli including pH value, temperature, light, redox potential, electric field, etc, by including a stimuli-sensitive compound in its structure (Yang & Kim, 2010; Ge et al., 2012; Guo & Kim, 2015; Moatsou et al., 2015; Lin et al., 2016; Abdollahi et al., 2019). Recently, ion pair self-assembly (IPSAM) was prepared using poly(ethyleneimine) (PEI) and (phenylthio)acetic acid (PTA) to obtain a temperature and oxidation-responsive drug carrier (Kim et al., 2021). PTA molecules could be ionically attached to PEI chains to form an ion pair because of the electrostatic attraction between the carboxyl groups of PTA and the amino groups of PEI. The ion pair was surface-active and amphiphilic and assembled into nanoparticles (i.e. IPSAM) in an aqueous phase. Moreover, IPSAM was temperature and oxidation-responsive in terms of its payload release. It was claimed that, when the temperature was raised, the ion pair lost its amphiphilic property due to an increased in PTA solubility and IPSAM was disintegrated, leading to a promoted release. In addition, it was also insisted that, under an oxidative condition, the ion pair lost its amphiphilic property due to the oxidation and the hydrophilization of PTA, resulting in the destabilization of IPSAM and an expedited release.

**Scheme 1.**
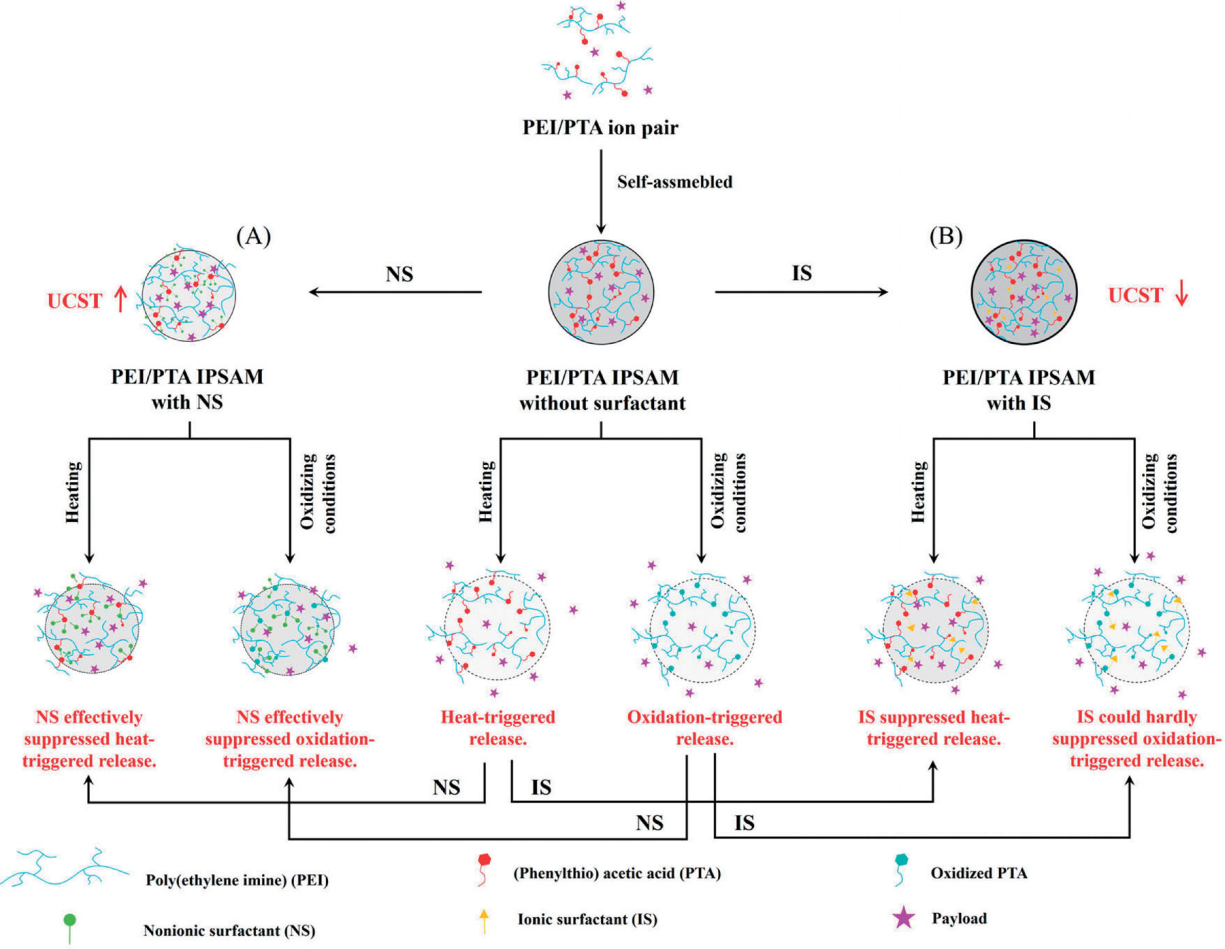
Effect of nonionic surfactant (A) and ionic surfactant (cationic and anionic surfactants) (B) on the UCST behavior of PEI/PTA ion pair and the temperature and oxidation-responsive release property of IPSAM.

In this study, the effect of surfactants on temperature-dependent assembling property of PEI/PTA ion pair were investigated using Brij S100 (BS100), cetylpridinium chloride (CPC), and N-lauroylsarcosine sodium salt (SLS) as a nonionic, a cationic, and an anionic surfactant, respectively. Surfactants are included in pharmaceutical preparations as an absorption enhancer, a dispersant, a solubilizer, etc. If PEI/PTA IPSAM is used as a drug carrier, the ion pair, the building block of the IPSAM, comes into contact with surfactants thus it may change its temperature–dependent assembling property in the pharmaceutical preparations. Thus it is worthwhile to examine the effect of the surfactants on the UCST behavior (i.e. the temperature-dependent assembling property) of the ion pair and the temperature–responsive release property of IPSAM. In addition, since PTA can be readily oxidized to the sulfoxide and the sulfone, the ion pair is susceptible to oxidation thus its assembly (IPSAM) may change its release property under an oxidizing condition, which can be given during the storage period of pharmaceutical preparations and in biological systems where IPSAM is administered into (Scheme 1).

## Experimental

### Materials

Poly(ethylene imine) (PEI, branched, average MW 2,000, 50 wt. % in H_2_O), Brij S100(>99%), cetylpridinium chloride (>98%), *N-*lauroylsarcosine sodium salt (>99%), phosphate buffer solution (PBS), and *N,N-d*imethylformamide (>99.8%) were purchased from Sigma-Aldrich Co. (St. louis, MO). (Phenylthio)acetic acid (PTA) (>97%) and nile red (>99%) were purchased from Tokyo Chemical Industry Co., Ltd. (Tokyo, Japan). Hydrogen peroxide (H_2_O_2_, 30%) was purchased from Daejung Chemicals & Metals Co., Ltd. (Siheung, South Korea). Water was doubly distilled in a Milli-Q water purification system (Millipore Corp, MA, USA) until the resistivity was 18 MΩ/cm. All other reagents were in analytical grade.

### Observation of upper critical solution temperature of PEI/PTA ion pair

10 mg of PEI was dissolved in 10 ml of PBS (10 mM, pH7.4) contained in a 20 ml vial, variable amounts of PTA (16.8, 39.1, and 90 mg) was added to PEI solution so that the molar ratio of amino to carboxyl group was 7:3, 5:5, and 3:7. The mixture suspensions were agitated at room temperature for 6 h using a roller mixer. 1.5 ml each of the suspensions was put in a cuvette, it was placed in a cuvette holder thermostated at a specific temperature in a range of 20–50 °C, and the transmittance was measured at 600 nm on a UV-Vis spectrophotometer (UK 6505 UV/Vis Spectrophotometer, JENWAY, UK). PEI/PTA mixture suspension whose the molar ratio of amino to carboxyl group was a:b was abbreviated to PEI/PTA(a/b) suspension. The ion pair and the IPSAM contained PEI/PTA(a/b) suspension were termed IP(a/b) and PEI/PTA(a/b), respectively. The upper critical solution temperature (UCST) was estimated using the intersection of two tangential lines connecting the data points in the constant region and in the rising region of the transmittance.

### Investigation of effect of surfactants on UCST of PEI/PTA ion pair

78.2 mg of PTA was dissolved in 20 ml of PEI solution (1 mg/ml, in PBS (10 mM, pH7.4)) so that the molar ratio of amino to carboxyl group was 5:5. Three kinds of surfactant (BS 100, CPC, and SLS) were separately added to the PEI/PTA(5/5) suspension so that the concentration of each surfactant was 0.05, 0.1, 0.2, 0.5, and 1.0 mM. PEI/PTA(5/5) suspension whose surfactant concentration was X mM was terms PEI/PTA(5/5)/surfactant name (X mM) suspension. The temperature-dependent transmittance of PEI/PTA(5/5)/surfactant name (X mM) suspension was determined as described in a previous section.

### Transmission electron microscopy

A negative staining technique was exploited to visualize IPSAM, following a method described elsewhere (Goldsmith & Miller, 2009; Scarff et al., 2018; Alle et al., 2021). Each of PEI/PTA(5/5), PEI/PTA(5/5)/BS 100(X mM), PEI/PTA(5/5)/CPC(X mM), and PEI/PTA(5/5)/SLS(X mM) suspension was mixed with a uranyl acetate solution (1.2% (w/v)) in equi-volume ratio and kept under dark condition at room temperature for 5 h. A formvar/copper-coated grid was put in the suspension so that IPSAM was deposited onto the surface of the grid and it was air-dried at room temperature overnight. The TEM micrograph of IPSAM was taken on a transmission electron microscope (LEO-912AB OMEGA, LEO, Germany), installed at Korea Basic Science Institute (KBSI, located in Chuncheon, Republic of Korea)).

### Observation of PEI/PTA/surfactants interaction by ^1^H NMR spectroscopy

PEI/PTA(5/5), PEI/PTA/(5/5)/BS 100(1 mM), PEI/PTA/(5/5)/CPC(1 mM), and PEI/PTA/(5/5)/SLS(1 mM) suspension were prepared as described previously except D_2_O was used as a solvent instead of the buffer solution. In parallel, each of PEI, PTA, BS100, CPC, and SLS was dissolved in D_2_O for the preparation of the single compound solution. ^1^H NMR spectrum was taken on a Bruker Avance 400 MHz spectrometer (Karlsruhe, Germany, installed in the Central Laboratory Center of Kangwon National University).

### Investigation of oxidation of PEI/PTA ion pair by X-ray photoelectron spectroscopy (XPS)

20 ml of PEI/PTA(5/5) suspension was prepared using a method described previously. H_2_O_2_ solution of different concentrations was added to the suspension contained in a 30 ml vial so that the final concentration of H_2_O_2_ was 0.005, 0.035, and 0.5%. The mixture suspensions were agitated at room temperature overnight using a roller mixer (205RM, Hwashin Technology Co., Korea) then they were freeze-dried. PTA, PEI/PTA(5/5)/H_2_O_2_(0.005%), PEI/PTA(5/5)/H_2_O_2_(0.035%), and PEI/PTA(5/5)/H_2_O_2_(0.5%) were subjected to XPS. The dry samples were put under the irradiation of a MaKa (1253.6 eV) achromatic X-ray (72 W) and the kinetic energy of atomic electrons was detected by a 180° double focusing hemispherical analyzer equipped with a 128-channel position-sensitive detector. The operation temperature was 20–23 °C and the pressure of a vacuum chamber was less than 5 × 1 0 ^−8^ mbar. The binding energy of C 1 s electron (284.5 eV) was used as the reference value to measure the binding energy of S 2p electron.

### Temperature-dependent release

100 μl of nile red solution (1 mg/ml, in DMF) was added to 10 ml each of PEI/PTA(5/5), PEI/PTA(5/5)/BS 100(0.1 mM), PEI/PTA(5/5)/CPC(0.1 mM), and PEI/PTA(5/5)/SLS(0.1 mM) suspension contained in a 20 ml vial. They were put on a roller mixer and agitated overnight at room temperature. 2 ml each of the suspensions was put in a cuvette and it was placed in a cuvette holder thermostated at the desired temperature. The fluorescence was measured at 615 nm using the excitation wavelength of 570 nm. The % release was determined using the following equation (Kim et al., 2021). % Release = (1 – F_t_/F_o_) X 100% where F_t_ was the fluorescence intensity at a given time and F_o_ was the initial fluorescence intensity. IPSAM contained in PEI/PTA(5/5)/BS 100(0.1 mM), PEI/PTA(5/5)/CPC(0.1 mM), and PEI/PTA(5/5)/SLS(0.1 mM) suspension were termed IPSAM(5/5)/BS 100(0.1 mM), IPSAM(5/5)/CPC(0.1 mM), and IPSAM(5/5)/SLS(0.1 mM), respectively.

### Oxidation-dependent release

100 μl of nile red solution (1 mg/ml, in DMF) was added to 10 ml each of PEI/PTA(5/5), PEI/PTA(5/5)/BS 100(0.1 mM), PEI/PTA(5/5)/CPC(0.1 mM), and PEI/PTA(5/5)/SLS(0.1 mM) suspension contained in a 20 ml vial. 0.4 ml of H_2_O_2_ solution with different concentrations was mixed with 1.6 ml each of the mixture suspensions so that H_2_O_2_ concentration was 0.005, 0.035 and 0.5%. All other conditions for the determination of % release were the same as those described in a previous section.

## Result and discussions

### Observation of UCST of PEI/PTA ion pair

**Figure 1.**
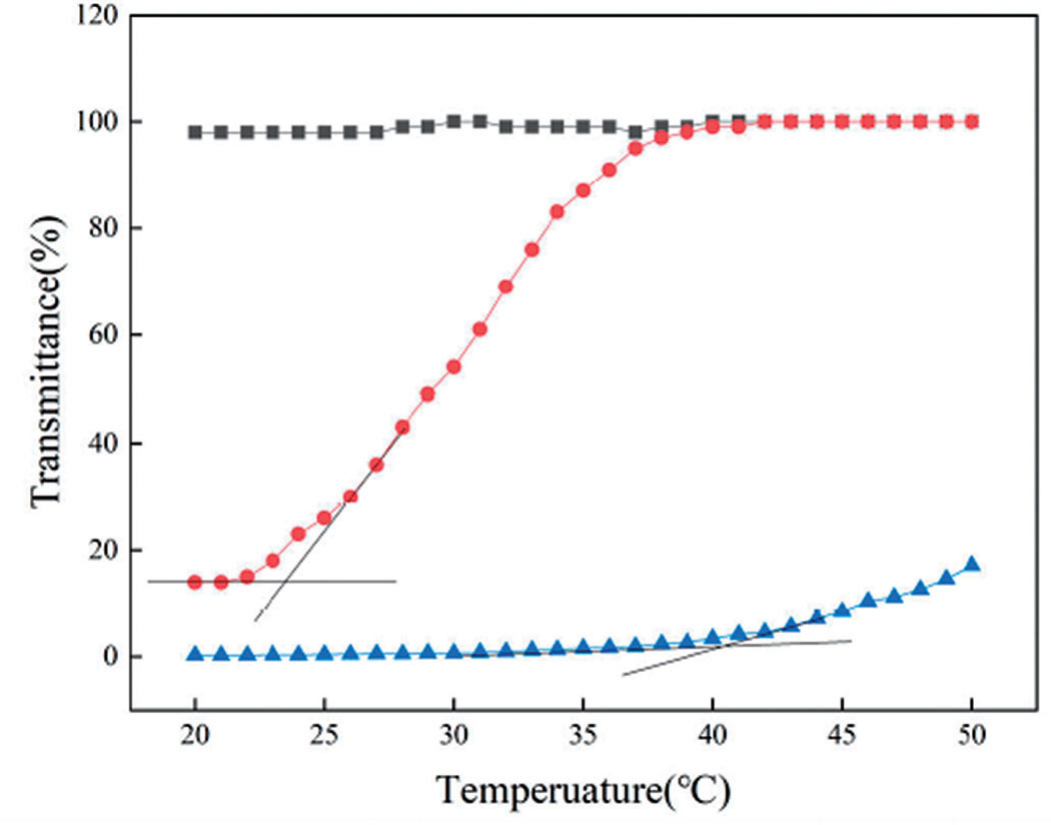
Temperature-dependent transmittance of PEI/PTA(3/7) (▲), PEI/PTA(5/5) (●), and PEI/PTA (7/3) (■) suspension.

Figure 1 shows the temperature-dependent transmittance of PEI/PTA(3/7), PEI/PTA(5/5), and PEI/PTA(7/3) suspension. PEI/PTA(3/7) suspension exhibited almost zero transmittance until the temperature reached 36 °C thereafter it increased in its transmittance with increasing the temperature. PTA molecules can be conjugated to PEI chains through an ionic interaction between the carboxyl group and the amino group. It was reported that the PEI/PTA ion pair was interface-active and amphiphilic and it was assembled into nanoparticles (i.e. IPSAM) in an aqueous solution (M. H. Wang et al., 2016). IPSAM can block visible light, leading to the zero of transmittance. As the suspension temperature increases by heating, PTA increases in its solubility in an aqueous solution and becomes hydrophilized, thus the PEI/PTA ion pair would lose its amphiphilic property, resulting in the disintegration of IPSAM. The increase in transmittance around 36 °C could be ascribed to the disintegration of IPSAM. Since the ion pair formed IPSAM in lower temperature range and it began to be dissolved at a certain temperature while being heated, the ion pair could be said to exhibit a UCST behavior. The UCST of PEI/PTA(3/7) ion pair was estimated to be about 40 °C by the intersection of two tangential lines. PEI/PTA(5/5) suspension exhibited almost constant transmittance below 23 °C thereafter it began to increase in its transmittance with increasing the temperature. The UCST of PEI/PTA(5/5) ion pair was estimated to be 20 °C, about 20 °C lower than that of PEI/PTA(3/7) ion pair. The lipophilicity of PEI/PTA ion pair would decrease as the content of PTA that contains the lipophilic phenyl group decreases. Thus, IPSAM composed of PEI/PTA with lower PTA content would require less thermal energy to be disintegrated. This could account for why PEI/PTA(5/5) ion pair showed UCST lower than that of PEI/PTA(3/7) ion pair. PEI/PTA(7/3) suspension exhibited almost 100% transmittance in the full range of temperature tested. Since PEI/PTA(7/3) ion pair has a small content of PTA and is lack of lipophilicity, it would hardly be assembled into IPSAM. The UCST of PEI/PTA(7/3) ion pair, if any, is likely to be out of the experimental temperature range. (i.e. below 20 °C).

### Investigation of effect of surfactants on UCST of PEI/PTA ion pair

**Figure 2.**
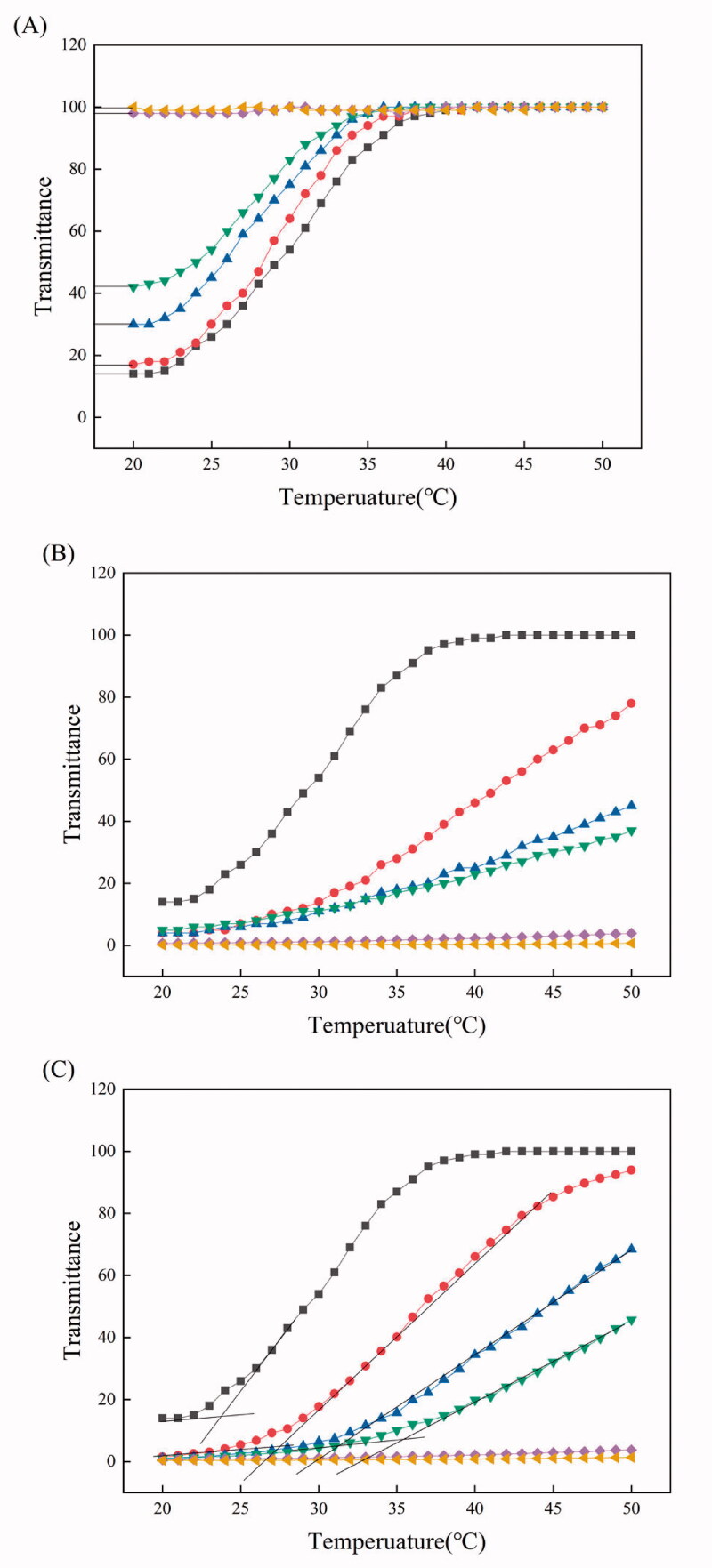
Temperature-dependent transmittance of PEI/PTA(5/5)/BS 100 (0 (■), 0.05 (●), 0.1 (▲), 0.2 (▼), 0.5 (◆), and 1.0 (◄) mM) suspension (A), PEI/PTA(5/5)/CPC (0 (■), 0.05 (●), 0.1 (▲), 0.2 (▼), 0.5 (◆), and 1.0 (◄) mM) suspension (B), and PEI/PTA(5/5)/SLS (0 (■), 0.05 (●), 0.1 (▲), 0.2 (▼), 0.5 (◆), and 1.0 (◄) mM) suspension (C).

Figure 2(A) shows the temperature-dependent transmittance of PEI/PTA(5/5)/BS 100 (X mM) suspension. As the concentration of BS 100 increased, the transmittance at a certain temperature increased. For example, when the concentration of BS 100 was 0, 0.05, 0.1, 0.2, 0.5, and 1.0 mM, the transmittance at 20 °C was about 14, 17, 30, 40, 98, and 100%, respectively. This suggested that the UCST of PEI/PTA(5/5) ion pair decreased with increasing the surfactant concentration. BS 100 is a kind of nonionic surfactant and it would hardly interact with PEI/PTA(5/5) ion pair through electrostatic interaction. Instead, it would be attracted to PTA of the ion pair through a hydrophobic interaction between the stearic group of the surfactant and the phenyl group of the aromatic compound (Figure 4(B)). In this circumstance, the nonionic surfactant is likely to surround PTA with its hydrophilic head (i.e. poly(ethylene oxide)) facing the aqueous bulk phase. Accordingly, the hydrophobic attraction among the phenyl groups of the PTA molecules of the ion pair would be weakened and the thermal energy required for the disintegration of IPSAM(5/5) (i.e. the dissolution of PEI/PTA(5/5) ion pair) would be reduced, leading to a decrease in UCST.

Figure 2(B) shows the temperature-dependent transmittance of PEI/PTA(5/5)/CPC (X mM) suspension. PEI/PTA(5/5)/CPC (X mM) suspension decreased in the increase rate of its transmittance when the concentration of CPC increased to 0.2 mM. Moreover, when the concentration was 0.5 and 1.0 mM, the suspension exhibited almost zero transmittance in the full range of temperature and hardly changed its transmittance with temperature. The UCST seemed to increase with increasing the concentration of CPC, which was opposite to the effect of BS 100 on the UCST. CPC is a cationic surfactant and its pyridinium group would be attached to the carboxyl group of PTA of PEI/PTA(5/5) ion pair through an electrostatic attraction (Figure 4(C)). As a result, CPC molecules can be electrostatically attached to PEI chains as hydrophobic pendants thus the hydrophobic interaction among the ion pairs and the intraparticle hydrophobic interaction of the resulting IPSAM (i.e. IPSAM(5/5)) would become stronger with increasing the concentration of CPC. Accordingly, when the concentration of CPC increases, PEI/PTA(5/5) ion pair would increase in its UCST, it would increase in its resistance against the thermal dissolution, and IPSAM(5/5) would increase in its resistance against the thermal disintegration.

Figure 2(C) shows the temperature-dependent transmittance of PEI/PTA(5/5)/SLS (X mM) suspension. As PEI/PTA(5/5)/CPC (X mM) suspension did, PEI/PTA(5/5)/SLS (X mM) suspension decreased in the increase rate of its transmittance as the concentration of SLS increased up to 0.2 mM. The suspension exhibited even almost zero transmittance at 0.5 and 1.0 mM in the full range of temperature tested and hardly changed its transmittance with temperature. That is, PEI/PTA(5/5) ion pair increased in its resistance against the thermal dissolution when the concentration of SLS increased, which was opposite to the effect of BS 100 on the UCST and similar to the effect of CPC on the UCST. SLS is an anionic surfactant and its sulfate group would be attached to the amino group of PEI of PEI/PTA(5/5) ion pair through an electrostatic attraction (Figure 4(D)). Consequently, SLS molecules can be conjugated to PEI chains as hydrophobic pendants thus the ion pairs would undergo a stronger intermolecular hydrophobic interaction and the resulting IPSAM would be subjected to a stronger intraparticle hydrophobic interaction when the concentration of SLS increases. This could account for why PEI/PTA(5/5) ion pair increased in its resistance against the thermal dissolution and IPSAM(5/5) increased in its resistance against the thermal disintegration when the concentration of SLS increased. Even though CPC is oppositely charged to SLS, the proposed mechanism by which the effect of CPC on the UCST could be explained was applicable to addressing a mechanism for the effect of SLS on the UCST.

### Transmission electron microscopy

**Figure 3.**
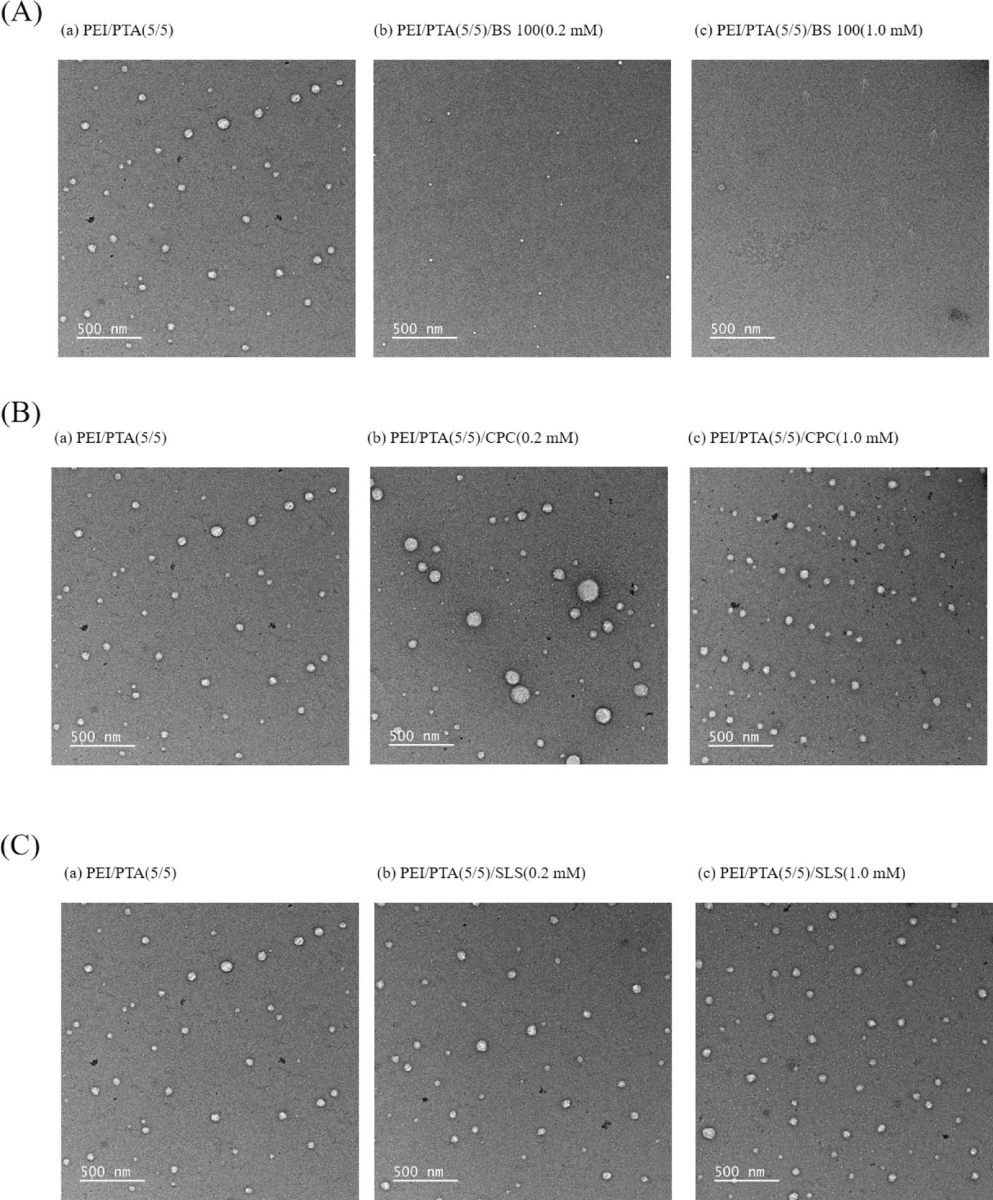
TEM micrograph of PEI/PTA(5/5) (a), PEI/PTA(5/5)/BS 100(0.2 mM) (b), and PEI/PTA(5/5)/BS 100(1.0 mM) (c) suspension (Arrows in (c) indicates tiny particles.) (A). TEM micrograph of PEI/PTA(5/5) (a), PEI/PTA(5/5)/CPC(0.2 mM) (b), and PEI/PTA(5/5)/CPC(1.0 mM) (c) suspension (B). TEM micrograph of PEI/PTA(5/5) (a), PEI/PTA(5/5)/SLS(0.2 mM) (b), and PEI/PTA(5/5)/SLS(1.0 mM) (c) suspension (C).

Figure 3(A) shows the TEM micrograph of PEI/PTA(5/5), PEI/PTA(5/5)/BS 100(0.2 mM), and PEI/PTA(5/5)/BS 100(1.0 mM) suspension. On the TEM micrograph of PEI/PTA(5/5) suspension, round particles were observed and they were less than 100 nm in diameter. PEI/PTA ion pair can be assembled into nanoparticles in an aqueous solution, the nanoparticles shown on the TEM micrograph were thought to be IPSAM. In fact, the transmittance of PEI/PTA(5/5) suspension was as low as 14% at room temperature (20–23 °C) and the UCST was close to that temperature (Figure 2(A)), suggesting that IPSAM(5/5) was contained in the suspension. On the TEM micrograph of PEI/PTA(5/5)/BS 100(0.2 mM) suspension, some smaller particles were found and they were 20–30 nm in diameter. The transmittance of PEI/PTA(5/5)/BS 100(0.2 mM) suspension was somewhat high (ca. 42%) at room temperature (20–23 °C) and the UCST seemed to be quite below that temperature (Figure 2(A)), indicating that IPSAM(5/5) was partially disintegrated by BS 100. The smaller particles shown on the TEM micrograph were thought to be partially disintegrated IPSAM(5/5). On the TEM micrograph of PEI/PTA(5/5)/BS 100(1.0 mM) suspension, tiny particles were found and they were less than 10 nm in diameter. The transmittance of PEI/PTA(5/5)/BS 100(1.0 mM) suspension was almost 100% at room temperature (20–23 °C) (Figure 2(A)), indicating that IPSAM(5/5) was totally disintegrated by BS 100. The tiny particles shown on the TEM micrograph were thought to be the micelle of BS 100. BS 100 would be hydrophobically attached to PTA of the ion pair to form micelles. A possible mechanism for the formation of micelles would be that the hydrophobic stearic group of the nonionic surfactant is likely to be hydrophobically attached to the hydrophobic phenyl group of PTA while its hydrophilic head (i.e. poly(ethylene oxide)) orienting toward aqueous bulk phase. Figure 3(B) shows the TEM micrograph of PEI/PTA(5/5)/CPC(0.2 mM) and PEI/PTA(5/5)/CPC(1.0 mM) suspension. On the TEM micrograph of PEI/PTA(5/5)/CPC(0.2 mM) suspension, particles were similar to IPSAM(5/5) contained in PEI/PTA(5/5) suspension in terms of the size and the shape. As described previously, CPC could be ionically bound to PTA and act as the hydrophobic pendants of PEI chains, resulting in an increase in the thermal stability of IPSAM(5/5) (Figure 2(B)). In fact, the transmittance of PEI/PTA(5/5)/CPC (0.2 mM) suspension was as low as 6% at room temperature (20–23 °C) and the UCST seemed to be higher than that temperature (Figure 2(B)), suggesting that IPSAM(5/5) was remained intact in the suspension. On the TEM micrograph of PEI/PTA(5/5)/CPC(1.0 mM) suspension, IPSAM(5/5) seemed to still remain intact with CPC(1.0 mM). In fact, the transmittance of PEI/PTA(5/5)/CPC(1.0 mM) suspension was almost zero at room temperature (20–23 °C) and the UCST was not found in the testing temperature range. This indicated that IPSAM(5/5) could hardly be disintegrated at the CPC concentration of 1.0 mM and remain stable in the suspension.

Figure 3(C) shows the TEM micrograph of PEI/PTA(5/5)/SLS(0.2 mM) and PEI/PTA(5/5)/SLS(1.0 mM) suspension. Nanoparticles shown on the TEM micrograph of PEI/PTA(5/5)/SLS(0.2 mM) suspension were not markedly different, in the size and the shape, from IPSAM(5/5) contained in PEI/PTA(5/5) suspension. As described previously, SLS would be ionically attached to PEI chains and play a role as the hydrophobic pendants of the polymer chains, thus it could increase the thermal stability of IPSAM(5/5) (Figure 2(C)). In fact, PEI/PTA(5/5)/SLS(0.2 mM) suspension exhibited almost zero % of the transmittance at room temperature (20–23 °C) and the UCST seemed to be higher than that temperature (Figure 2(C)). This suggested that IPSAM(5/5) was stable in presence of SLS (0.2 mM) at room temperature (20–23 °C). Nanoparticles shown on the TEM micrograph of PEI/PTA(5/5)/SLS(1.0 mM) suspension were also apparently similar to IPSAM(5/5) contained in PEI/PTA(5/5) suspension. PEI/PTA(5/5)/SLS(1.0 mM) suspension showed almost zero % of the transmittance at room temperature (20–23 °C) and it did not show any UCST behavior in the testing temperature range (Figure 2(C)). This suggested that IPSAM(5/5) maintained its integrity in presence of SLS (1.0 mM) at room temperature (20–23 °C).

## Observation of PEI/PTA/surfactants interaction by ^1^H NMR spectroscopy

Figure 4(A) shows the ^1^H NMR spectrum of PEI, PTA, and PEI/PTA(5/5). The phenyl group of PTA was found around 7.45 ppm in the spectrum of PTA. The phenyl group of PTA was found around 7.33 ppm in the spectrum of IPSAM(5/5). As described previously, IPSAM was thought to be formed due to the hydrophobic interaction among the phenyl groups of PEI/PTA ion pairs. The up-field shift could be ascribed to the hydrophobic interaction. Figure 4(B) shows the ^1^H NMR spectrum of BS 100, PEI/PTA(5/5), and PEI/PTA/(5/5)/BS 100(1 mM). In the spectrum of PEI/PTA/(5/5)/Brij(1 mM), the signal position of the phenyl group hardly changed, but a very small new peak appeared on the left shoulder. The stearic group of BS 100 would be able to be attracted to the phenyl group of PTA by a hydrophobic interaction, which may deform the electron cloud of the phenyl group.

Figure 4.^1^H NMR spectrum of PEI (a), PTA (b), and PEI/PTA(5/5) (c) (A). ^1^H NMR spectrum of BS 100 (a), PEI/PTA(5/5) (b), and PEI/PTA(5/5)/BS 100(1 mM) (c) (B). ^1^H NMR spectrum of CPC (a), PEI/PTA(5/5) (b), and PEI/PTA(5/5)/CPC(1 mM) (c) (C). ^1^H NMR spectrum of SLS (a), PEI/PTA(5/5) (b), and PEI/PTA(5/5)/SLS(1 mM) (c) (D).

**Figure:**
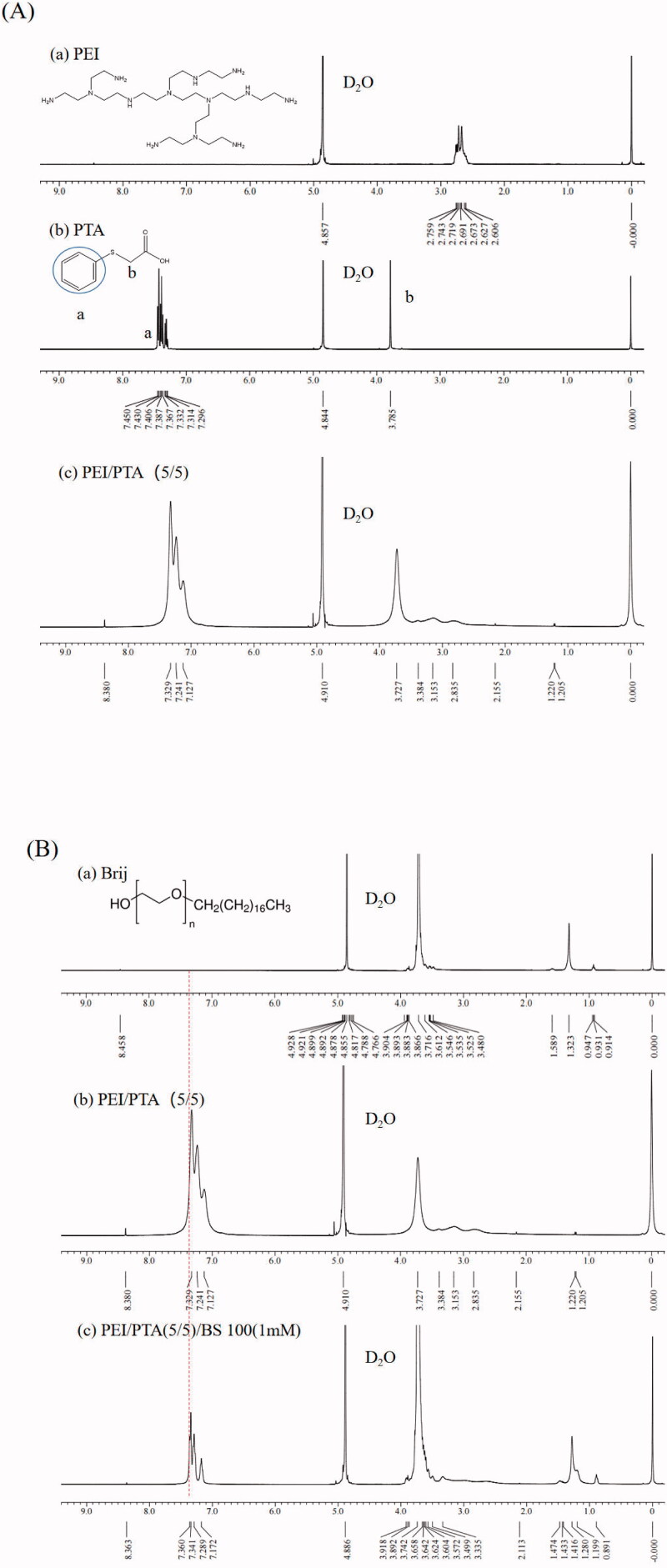

**Figure:**
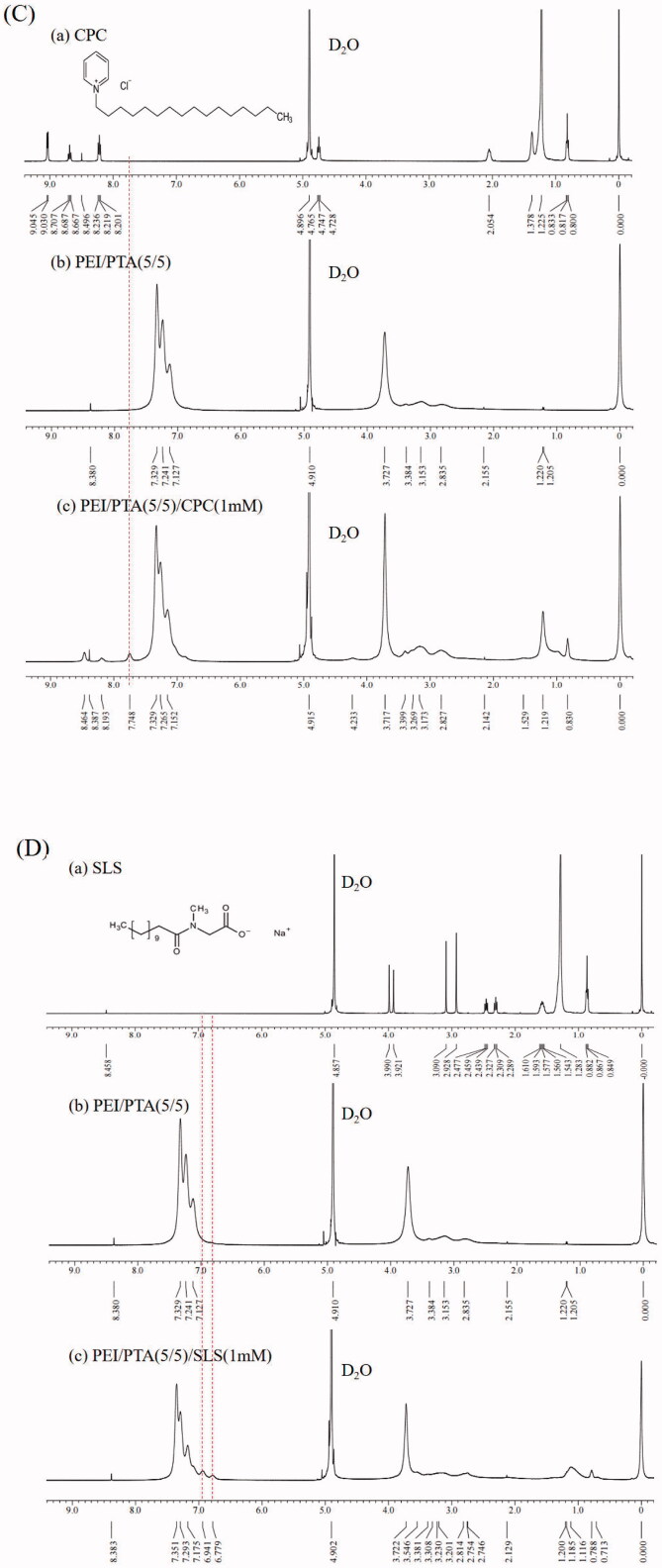

Figure 4(C) shows the ^1^H NMR spectrum of CPC, PEI/PTA(5/5), and PEI/PTA/(5/5)/CPC(1 mM). In the spectrum of PEI/PTA/(5/5)/CPC(1 mM), the peak at the main position of the phenyl group did not change, but a new peak appeared at 7.748 ppm. The emergence of this new peak was possibly because the pyridinium group of CPC could electrostatically interact with the carboxyl group of PTA, affecting the electron distribution of the phenyl group. Figure 4(D) shows the ^1^H NMR spectrum of SLS, PEI/PTA(5/5), and PEI/PTA/(5/5)/SLS(1 mM). Like the spectrum of PEI/PTA/(5/5)/CPC(1 mM), the main peak of phenyl group did not change, but new peaks were found at 6.941 and 6.779 ppm on the right side of the main peak on the contrary to the spectrum of PEI/PTA/(5/5)/CPC(1 mM). The sulfate group of SLS would electrostatically interact with the amino group of PEI. Accordingly, SLS can compete with PTA when binding to PEI electrostatically, thus PTA would decrease in its chance to attach to the cationic polymer chains, which may affect the electron cloud distribution of the phenyl group.

### Investigation of oxidation of PEI/PTA ion pair by XPS

**Figure 5.**
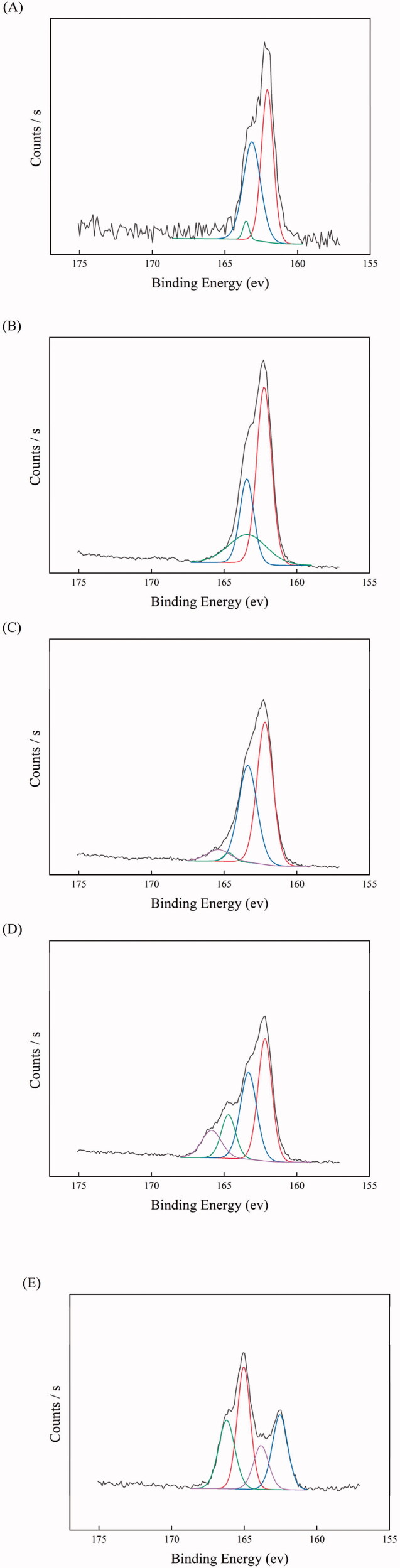
XPS spectrum of PTA (A), PEI/PTA(5/5) (B), PEI/PTA(5/5)/H_2_O_2_(0.005%) (C), PEI/PTA(5/5)/H_2_O_2_(0.035%) (D), and PEI/PTA(5/5)/H_2_O_2_(0.5%) (E).

Figure 5 shows the XPS spectrum of the S atom signal of PTA, PEI/PTA(5/5), PEI/PTA(5/5)/H_2_O_2_(0.005%), PEI/PTA(5/5)/H_2_O_2_(0.035%), and PEI/PTA(5/5)/H_2_O_2_(0.5%). In the spectrum of PTA (Figure 5(A)) and PEI/PTA(5/5) (Figure 5(B)), the sulfide peak of PTA was found around 163 eV. In the spectrum of PEI/PTA(5/5)/H_2_O_2_(0.005%) (Figure 5(C)) and PEI/PTA(5/5)/H_2_O_2_(0.035%) (Figure 5(D)), the signal of the sulfoxide and the sulfone group were found around 165 eV and 167 eV, respectively, along with the sulfide signal around 163 eV. In the spectrum of PEI/PTA(5/5)/H_2_O_2_(0.5%) (Figure 5(E)), the signal of the sulfone group was found but not that of the sulfoxide group, suggesting that the oxidation degree was higher at a higher concentration of the oxidant. From the results described above, it could be said that the PTA of PEI/PTA IPSAM could be readily oxidized by H_2_O_2_ even at a low concentration (e.g. 0.005%).

### Temperature-dependent release

**Figure 6.**
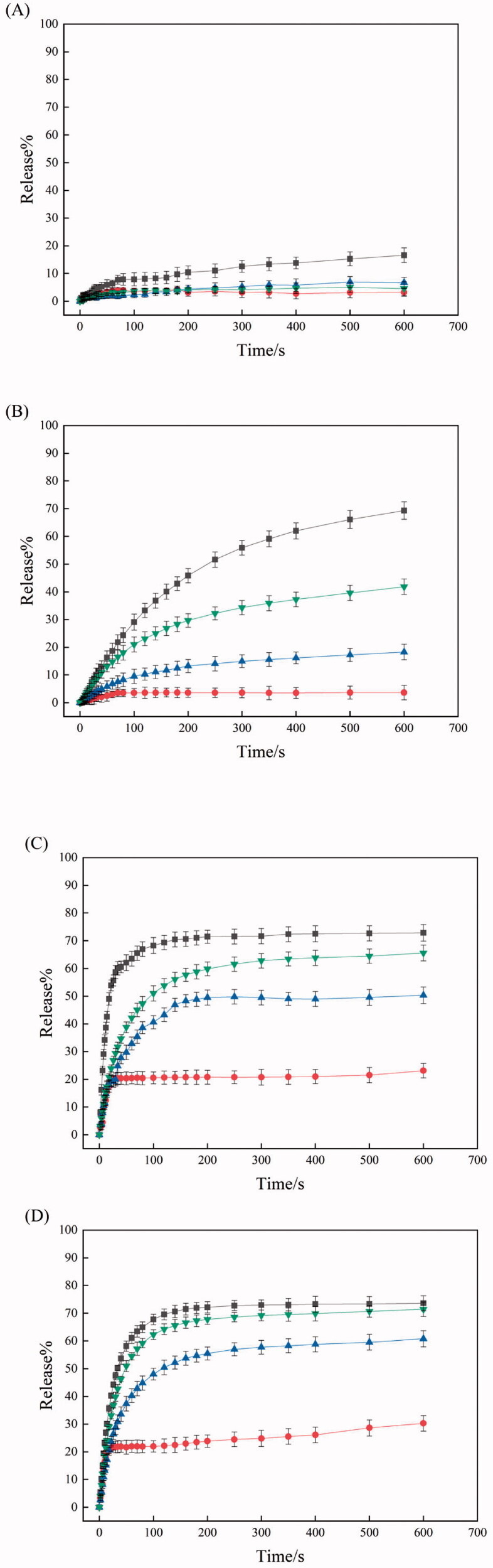
Release profile at 24 °C (A), 30 °C (B), 37 °C (C), and 45 °C (D) of dye loaded in IPSAM (5/5) (■), IPSAM(5/5)/BS 100(0.1 mM) (●), IPSAM(5/5)/CPC(0.1 mM) (▲), and IPSAM(5/5)/SLS(0.1 mM) (▼).

Figure 6(A) shows the release profile at 24 °C of dye loaded in IPSAM (5/5), IPSAM(5/5)/BS 100(0.1 mM), IPSAM(5/5)/CPC(0.1 mM), and IPSAM(5/5)/SLS(0.1 mM). The release degree of dye loaded in IPSAM(5/5) increased to about 17% in 600 s. Since the UCST of IPSAM (5/5) was above 23 °C, the IPSAM can be partially disintegrated at the medium temperature (i.e. 24 °C), giving rise to a significant release. IPSAM(5/5)/CPC(0.1 mM) exhibited a suppressed release and the maximum release degree of dye loaded in the IPSAM was only 4.5%, lower than that of dye loaded in IPSAM (5/5). This could be ascribed to that CPC could increase the thermal stability of the IPSAM (Figure 2(B)). IPSAM(5/5)/SLS(0.1 mM) also showed a suppressed release and the maximum release degree was only 7%, possibly because of the IPSAM-stabilizing effect of SLS (Figure 2(C)). So did IPSAM(5/5)/BS 100(0.1 mM) and the maximum release degree was only 3%. Unlike CPC and SLS, BS 100 decreased the UCST of PEI/PTA(5/5) ion pair (Figure 2(A)), deteriorated the thermal stability of the IPSAM, and partially disintegrated it (Figure 3(A)). Nevertheless, the release of cargo loaded in IPSAM(5/5)/BS 100 was suppressed. As described previously, BS 100 would be able to form micelles around PTA molecules and it could take up the lipophilic dye (i.e. nile red). The micellization would be a reason why the release was suppressed despite the IPSAM was disintegrated by BS 100.

Figure 6(B) shows the release profile at 30 °C of dye loaded in IPSAM (5/5), IPSAM(5/5)/BS 100(0.1 mM), IPSAM(5/5)/CPC(0.1 mM), and IPSAM(5/5)/SLS(0.1 mM). The release degree increased following a saturation trajectory and the release degree was greater in the order of IPSAM (5/5) > IPSAM(5/5)/SLS(0.1 mM) > IPSAM(5/5)/CPC(0.1 mM) > IPSAM(5/5)/BS 100(0.1 mM). For example, the maximum release degree of dye loaded in IPSAM (5/5), IPSAM(5/5)/SLS(0.1 mM), IPSAM(5/5)/CPC(0.1 mM), and IPSAM(5/5)/BS 100(0.1 mM) were about 69.3, 41.8, 18.3, and 3.7%, respectively. The elaboration on the release property of each IPSAM at 24 °C was also applicable to explaining the release property at 30 °C. The release degree of dye loaded in IPSAM(5/5)/SLS(0.1 mM) and IPSAM(5/5)/CPC(0.1 mM) was lower than that of dye loaded in IPSAM(5/5). Owing to the UCST-increasing effect of CPC and SLS (Figure 2(B,C)), IPSAM(5/5) would be stabilized by the ionic surfactants and the release of cargo loaded in the IPSAM could be suppressed. The release degree of dye loaded in IPSAM(5/5)/CPC(0.1 mM) was lower than that of dye loaded in IPSAM(5/5)/SLS(0.1 mM). Since CPC could slacken down the slope of the transmittance profile of the suspension more effectively than SLS, it could be said that IPSAM-stabilizing effect of the cationic surfactant was higher than that of the anionic one. This could account for why CPC could suppress the release more effectively. IPSAM(5/5)/BS 100(0.1 mM) exhibited the lowest release degree even though its thermal stability was the weakest among the samples tested. This discrepancy could be explained by the micellization of the nonionic surfactant. Except for IPSAM(5/5)/BS 100(0.1 mM), the IPSAMs released their payload much more at 30 °C than at 24 °C. The medium temperature of 24 °C was around or less than the UCST of the ion pairs thus it could allow the IPSAMs to release only a small amount of their content. But, the medium temperature of 30 °C was obviously higher than the UCST thus it could make the IPSAMs release more extensively. Figure 6(C) shows the release profile at 37 °C of dye loaded in IPSAM (5/5), IPSAM(5/5)/BS 100(0.1 mM), IPSAM(5/5)/CPC(0.1 mM), and IPSAM(5/5)/SLS(0.1 mM). Like the release at 30 °C, the release degree at 37 °C also increased in a saturation manner and the order of the release degree was the same as that observed at 30 °C (i.e. IPSAM (5/5) > IPSAM(5/5)/SLS(0.1 mM) > IPSAM(5/5)/CPC(0.1 mM) > IPSAM(5/5)/BS 100(0.1 mM)). As described above, since CPC and SLS could increase the UCST (Figure 2(B,C)), IPSAM(5/5)/SLS(0.1 mM) and IPSAM(5/5)/CPC(0.1 mM) would be thermally more stable than IPSAM (5/5), accounting for why the IPSAMs containing the ionic surfactants released their content less than the IPSAM without the surfactants. Since the IPSAM-stabilizing effect of the cationic surfactant was higher than that of the anionic one (Figure 2(B,C)), the thermal stability of IPSAM(5/5)/CPC(0.1 mM) would be stronger than that of IPSAM(5/5)/SLS(0.1 mM), accounting for why the IPSAM containing the cationic surfactant released its cargo less than the IPSAM containing the anionic surfactant. Despite of the weakest thermal stability, IPSAM(5/5)/BS 100(0.1 mM) showed the lowest release degree, possibly due to the micellization of the nonionic surfactant. The initial release rate and the release degree of dye loaded in each IPSAM at 37 °C were higher than those observed at 30 °C (Figure 6(B,C)). For example, the maximum release degree at 37 °C of dye loaded in IPSAM (5/5), IPSAM(5/5)/SLS(0.1 mM), IPSAM(5/5)/CPC(0.1 mM), and IPSAM(5/5)/BS 100(0.1 mM) were about 72.5, 64, 50, and 23%, respectively (Figure 6(C)), which were higher than the corresponding maximum values at 30 °C (Figure 6(B)). As the temperature of the release medium deviates from the UCST more, the IPSAM would be more readily disintegrated and release more amount of its cargo.

Figure 6(D) shows the release profile at 45 °C of dye loaded in IPSAM (5/5), IPSAM(5/5)/BS 100(0.1 mM), IPSAM(5/5)/CPC(0.1 mM), and IPSAM(5/5)/SLS(0.1 mM). The release profiles observed at 45 °C resembled those at 37 °C. The IPSAMs released their content slightly more at the higher temperature than at the lower temperature. For example, the maximum release degree at 37 °C of dye loaded in IPSAM (5/5), IPSAM(5/5)/SLS(0.1 mM), IPSAM(5/5)/CPC(0.1 mM), and IPSAM(5/5)/BS 100(0.1 mM) were about 73, 71, 60, and 30%, respectively (Figure 6(D)), which were slightly higher than the corresponding maximum values at 37 °C (Figure 6(C)). Since the disintegration of IPSAM can be thermally expedited, the higher the temperature is, the more the release would take place. However, the release degree at 37 °C was not markedly different from that at 45 °C. Hence, it could be said the temperature of 37 °C was high enough to cause the IPSAM to release its content sufficiently.

### Oxidation-dependent release

**Figure 7.**
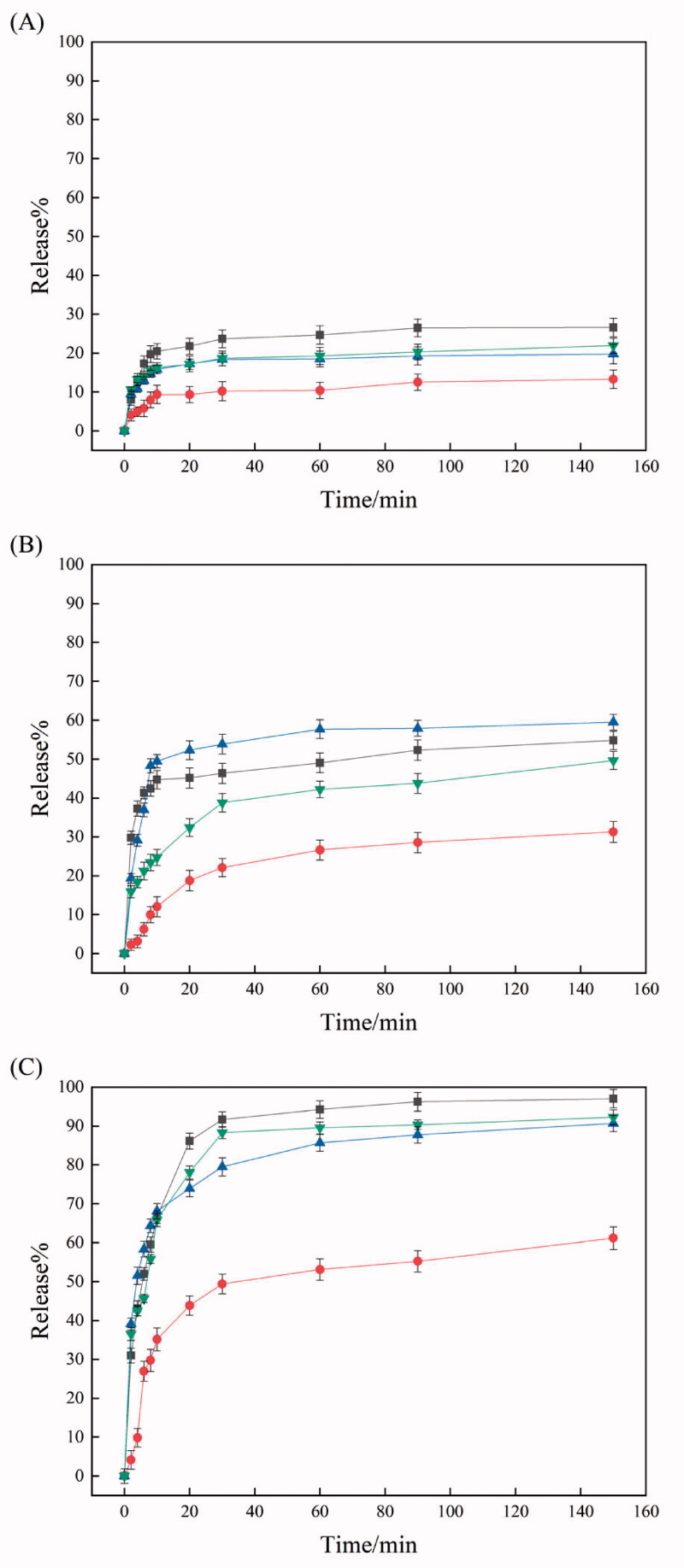
Release profile of dye loaded in IPSAM (5/5) (■), IPSAM(5/5)/BS 100(0.1 mM) (●), IPSAM(5/5)/CPC(0.1 mM) (▲), and IPSAM(5/5)/SLS(0.1 mM) (▼) when H_2_O_2_ concentration was 0.005% (A), 0.035% (B), and 0.5% (C).

Figure 7(A) shows the release profile of dye loaded in IPSAM (5/5), IPSAM(5/5)/BS 100(0.1 mM), IPSAM(5/5)/CPC(0.1 mM), and IPSAM(5/5)/SLS(0.1 mM) when H_2_O_2_ concentration was 0.005%. The release degree at H_2_O_2_ concentration of 0.005% was higher than that without H_2_O_2_ (Figures 6(A) and 7(A)). For example, the maximum release degree of dye loaded in IPSAM (5/5), IPSAM(5/5)/BS 100(0.1 mM), IPSAM(5/5)/CPC(0.1 mM), and IPSAM(5/5)/SLS(0.1 mM) was about 27, 22, 20, and 13%, which was significantly higher than the corresponding maximum release degree obtained without H_2_O_2_. A sulfide compound is known to be oxidized to a sulfoxide and a sulfone (Ta & Porter, 2013). Thus, PTA could be oxidized to the oxidized compounds by the oxidizing agent (i.e. H_2_O_2_) and become more polar. As a result, PTA, the hydrophobic pendant of PEI chains, would decrease in its hydrophobicity thus the hydrophobic attraction among PEI/PTA ion pairs and the intraparticle hydrophobic attraction of IPSAM would be weakened, leading to the disintegration of the nanoparticles. This could account for why the release degree was promoted by H_2_O_2_. IPSAM (5/5) released its content somewhat more than IPSAM(5/5)/CPC(0.1 mM) and IPSAM(5/5)/SLS(0.1 mM). The IPSAMs seemed to reflect the UCST and the thermal stability in releasing their content even after being oxidized when H_2_O_2_ concentration was low (e.g. 0.005%) (The UCST of PEI/PTA(5/5) ion pair without an ionic surfactant was lower than that of the ion pair with an ionic surfactant (Figure 2(B,C)). On the other hand, IPSAM(5/5)/BS 100(0.1 mM) released its cargo significantly less than the IPSAMs with an ionic surfactant even though its thermal stability was the lowest among the testing IPSAMs. This is possible because BS 100 would be assembled around the phenyl group of PTA to form micelles, which could accommodate nile red released from the IPSAM.

Figure 7(B) shows the release profile of dye loaded in IPSAM (5/5), IPSAM(5/5)/BS 100(0.1 mM), IPSAM(5/5)/CPC(0.1 mM), and IPSAM(5/5)/SLS(0.1 mM) when H_2_O_2_ concentration was 0.035%. The release degree at H_2_O_2_ concentration of 0.035% was higher than that obtained at H_2_O_2_ concentration of 0.005% (Figure 7(A,B)). For example, the maximum release degree of dye loaded in IPSAM (5/5), IPSAM(5/5)/BS 100(0.1 mM), IPSAM(5/5)/CPC(0.1 mM), and IPSAM(5/5)/SLS(0.1 mM) were about 59.5, 55, 50, and 31%, respectively, which were much higher than the corresponding maximum release degree obtained at H_2_O_2_ concentration of 0.005%. As H_2_O_2_ concentration was higher, PTA would be oxidized more extensively, the hydrophobic attraction among PEI/PTA ion pairs would be weakened more, thus the IPSAMs would be disintegrated more readily, leading to a higher release degree. There was no marked difference in the release degree among the IPSAMs except for IPSAM(5/5)/BS 100(0.1 mM) (Figure 7(B)). The IPSAMs seemed not to reflect the UCST and the thermal stability in releasing their content after being oxidized when H_2_O_2_ concentration was 0.035%. Once PTA is oxidized to some degrees (Figure 5), the oxidization-induced hydrophilization of PTA would overwhelm the lipophilization of PEI caused by the ionic surfactants, which could make no difference in the thermal stability of the IPSAMs. Meanwhile, the release degree of dye loaded in IPSAM(5/5)/BS 100(0.1 mM) was exceptionally low, possibly due to the micellization of the nonionic surfactant.

Figure 7(C) shows the release profile of dye loaded in IPSAM (5/5), IPSAM(5/5)/BS 100(0.1 mM), IPSAM(5/5)/CPC(0.1 mM), and IPSAM(5/5)/SLS(0.1 mM) when H_2_O_2_ concentration was 0.5%. All the IPSAMs released their content more extensively at H_2_O_2_ concentration of 0.5% than at 0.035%. For example, IPSAM (5/5), IPSAM(5/5)/BS 100(0.1 mM), IPSAM(5/5)/CPC(0.1 mM), and IPSAM(5/5)/SLS(0.1 mM) showed their maximum release degree of about 97, 95.9, 95.1, and 61%, respectively. The maximum values were significantly higher than those obtained at H_2_O_2_ concentration of 0.035% (Figure 7(B,C)), possibly because PTA can be oxidized more readily at a higher oxidizing agent concentration. IPSAM(5/5)/BS 100(0.1 mM) still exhibited a relatively low release degree among the IPSAMs tested and the micelles of BS 100 were supposed to incorporate the dye release out of the IPSAM into their hydrophobic cores. Since all the IPSAMs except for IPSAM(5/5)/BS 100(0.1 mM) exhibited almost 100% release, H_2_O_2_ concentration of 0.5% was thought to be enough to disintegrate the IPSAMs completely.

## Conclusions

BS 100 (a nonionic surfactant) decreased the UCST of PEI/PTA ion pair, but CPC (a cationic surfactant) and SLS (an anionic surfactant) increased it. Due to the hydrophobic interaction between BS 100 and PTA, the hydrophobic interaction among the phenyl groups of the PTA molecules of the ion pair would be weakened, leading to a decrease in UCST. Owing to the electrostatic interaction between CPC and PTA and between SLS and PEI, CPC and SLS would play a role as the hydrophobic pendants of the polymer chains, resulting in an increase in UCST. According to the TEM microscopy, BS 100 reduced the size of IPSAM markedly whereas CPC and SLS hardly affected the size and the integrity of IPSAM, which was in accordance with the UCST behavior of PEI/PTA ion pair in presence of the surfactants. ^1^H NMR spectroscopy revealed the hydrophobic interaction among the phenyl groups of PEI/PTA ion pairs took place, accounting for a mechanism of IPSAM formation. It also evidenced the hydrophobic interaction between BS 100 and PTA and the electrostatic interaction between CPC and PTA and between SLS and PEI occurred, which could explain the effect of the surfactants on the UCST of PEI/PTA ion pair. According to XPS spectroscopy, the PTA of IPSAM could be readily oxidized by H_2_O_2_ even at a low concentration (e.g. 0.005%) and the oxidation degree increased with the oxidant concentration. IPSAM exhibited a temperature and oxidation-responsive release of its payload (i.e. nile red). The surfactants suppressed the temperature-responsive release effectively in the order of BS 100 > CPC > SLS. BS 100 would be assembled into micelles that could uptake the payload released out of IPSAM, rendering the release degree lowered. The ionic surfactants (i.e. CPS and SLS) could be ionically attached to PEI chains and strengthen the hydrophobic interaction among PEI/PTA ion pairs, leading to a suppressed release. The IPSAMs released their content more extensively as H_2_O_2_ concentration was higher, suggesting that they were oxidation-responsive in terms of release. Once PTA was oxidized to some degrees at a high oxidizing agent concentration (e.g. 0.035%), there was no marked difference in the release degree among the IPSAMs except for IPSAM(5/5)/BS 100(0.1 mM). The ionic surfactants hardly affected the oxidation-induced release degree but the nonionic surfactant (BS 100) suppressed the release degree significantly, possibly due to the micellization. The results obtained in this study would be useful to understand the temperature-dependent assembling behavior of the ion pair and the temperature and oxidation-responsive release property of IPSAM in surfactant-containing preparations (e.g. pharmaceutics and cosmetics).
